# Prevalence and Genetic Analysis of Chromosomal *mcr-3/7* in *Aeromonas* From U.S. Animal-Derived Samples

**DOI:** 10.3389/fmicb.2021.667406

**Published:** 2021-04-30

**Authors:** Yan Wang, Naxin Hou, Reuven Rasooly, Yongqiang Gu, Xiaohua He

**Affiliations:** ^1^Western Regional Research Center, Agricultural Research Service, United States Department of Agriculture, Albany, CA, United States; ^2^State Key Laboratory of Infectious Disease Prevention and Control, National Institute for Communicable Disease Control and Prevention, Chinese Center for Disease Control and Prevention, Beijing, China

**Keywords:** *Aeromonas*, antimicrobial resistance, colistin resistance, food-producing animals, prevalence, whole genome sequence

## Abstract

The prevalence of *mcr*-positive bacteria in 5,169 domestic animal-derived samples collected by USDA Food Safety and Inspection Service between October 2018 and May 2019 was investigated. A procedure including enriched broth culture and real-time PCR targeting *mcr-1* to *mcr-8* were used for the screening. Fifteen positive isolates were identified, including one plasmid-borne *mcr-1*-positive *Escherichia coli* strain, EC2492 (reported elsewhere) and 14 *mcr-3/7*-positive strains from poultry (1), catfish (2), and chicken rinse (11) samples, resulting in an overall prevalence of *mcr*-positive bacteria 0.29% in all meat samples tested. Analysis of 16S rRNA and whole genome sequences revealed that all 14 strains belonged to *Aeromonas*. Data from phylogenetic analysis of seven housekeeping genes, including *gyrB, rpoD, gyrA, recA, dnaJ, dnaX*, and *atpD*, indicated that nine strains belonged to *Aeromonas hydrophila* and five strains belonged to *Aeromonas jandaei*. Antimicrobial tests showed that almost all *mcr*-positive strains exhibited high resistance to colistin with MICs ≥ 128mg/L, except for one *A. jandaei* strain, which showed a borderline resistance with a MIC of 2 mg/L. A segment containing two adjacent *mcr-3* and *mcr-3-*lik*e* genes was found in two *A. hydrophila* and one *A. jandaei* strains and a variety of IS-like elements were found in the flanking regions of this segment. A *mcr-3*-related lipid A phosphoethanolamine transferase gene was present in all 14 *Aeromonas* strains, while an additional *mcr-7*-related lipid A phosphoethanolamine transferase gene was found in 5 *A. jandaei* strains only. In addition to *mcr* genes, other antimicrobial resistance genes, including *bla*_OXA–12/OXA–724_, *aqu-2, tru-1*, *cepS*, *cphA*, *imiH*, *ceph-A3*, *ant(3″)-IIa*, *aac(3)-Via*, and *sul1* were observed in chromosomes of some *Aeromonas* strains. The relative high prevalence of chromosome-borne *mcr-3/7* genes and the close proximity of various IS elements to these genes highlights the need for continued vigilance to reduce the mobility of these colistin-resistance genes among food animals.

## Introduction

The polymyxins, including polymyxin B and polymyxin E (colistin), are a group of cationic lipopeptide antibiotics against most Gram-negative bacteria by interacting with lipid A to disrupt the outer membrane and cause cell death (Falagas and Rafailidis, 2008). It was discontinued for routine use in humans due to its kidney toxicity. The increased emergence of multidrug resistant (MDR) bacteria has caused the reintroduction of polymyxins as the last-resort antibiotics in clinic. However, the recent discovery of mobile colistin resistance genes (*mcr-1* to *mcr-10*) in a broad range of sources has jeopardized the clinical efficacy of colistin (Liu et al., 2016; Xavier et al., 2016; Borowiak et al., 2017; Carattoli et al., 2017; Yin et al., 2017; AbuOun et al., 2018; Wang et al., 2018; Yang et al., 2018; Carroll et al., 2019; Wang C. et al., 2020).

To minimize further spread of colistin resistance, investigations on the prevalence of *mcr-*positive bacteria from humans, retail meat, and environmental samples have been performed globally (Luo et al., 2020). However, reports on systemic screening of *mcr*-positive bacteria in animal-derived food products in the United States were rare.

In this study, we screened 5,169 food-producing animal samples including chicken rinse, ground beef, beef trim, pork, poultry, and catfish collected by the Food Safety and Inspection Service (FSIS, USDA) to investigate the prevalence of *mcr*-positive Gram-negative bacteria; characterize *mcr*-positive bacterial strains based on the analysis of whole genome sequences; identify *mcr* variants and analyze their genetic environment in bacterial genomes.

## Materials and Methods

### Sample Collection, Processing, and Analysis

A total of 5,169 samples, including 1,787 chicken rinses, 1,369 ground beef, 1,057 beef trim, 416 pork, 363 poultry, and 177 catfish, randomly collected at various locations across the U.S. between October 10, 2018 and May 10, 2019, were sent to the FSIS Western Laboratory and processed as described, previously (Wang Y. et al., 2020). Briefly, samples were subjected to a non-selective enrichment at 35 or 42°C overnight with one part meat in three parts media. A neutralizing Buffered Peptone Water (nBPW) was used for chicken rinse samples and modified Tryptone-Soy Broth (mTSB) was used for all other samples. Overnight cultures (200 μL) were then transferred to a 96 deep-well block pre-filled with 800 μl/well of TSB selection medium containing CaCl_2_ (5 mM), colistin (4 μg/mL) and vancomycin (50 μg/mL, to reduce Gram-positive bacteria) and further incubated overnight at 37°C. These samples constitute approximately 14–29% of each sample type analyzed by FSIS during this time period according to FSIS Annual Sampling Program Plan at https://www.fsis.usda.gov/sites/default/files/media_file/2021-02/fsis-annual-sampling-plan-fy2021.pdf (accessed on 3/25/2021). A listing of FSIS Inspected Establishments can be found at https://www.fsis.usda.gov/inspection/establishments/meat-poultry-and-egg-product-inspection-directory (accessed on 3/25/2021).

### Identification and Isolation of *mcr*-positive Bacteria

Following enrichments of samples in non-selective and then selective media, wells with visible bacterial growth were analyzed by real-time PCR targeting on *mcr-1* to *mcr-8* using the Rotor-Gene Q (Qiagen) system. A total of five pairs of primers were designed for amplification of *mcr-1* to *mcr-8* fragments (Table 1). The primers for *mcr-1/2/6* were designed based on the alignment of *mcr-1.1* (NG_050417), *mcr-2.1* (NG_051171), and *mcr-6.1* (MF176240). The primers for *mcr-3/7* were designed based on the alignment of *mcr-3.1* (NG_056184) and *mcr-7.1* (NG_056413). The primers for *mcr-4*, *mcr-5*, and *mcr-8* were designed based on the *mcr-4.1* (MG459156), *mcr-5.1* (NG_055658) and *mcr-8.1* (NG_061399) genes, respectively. All primers listed in Table 1 were designed using Primer-BLAST, and the specificity of each pair of primers was evaluated using the Basic Local Alignment Search Tool (BLAST) on NCBI. For real-time PCR analysis, cells from 100 μL of overnight cultures were collected by centrifugation and resuspended in 100 μL of water and then lysed by boiling for 10 min. After removing cell debris by centrifugation, the clear lysate was used as DNA template. A 20 μL of PCR reaction included 2 μL of cell lysate (∼100 ng DNA template), 0.5 μM of each primer, 1x SYBR Green Master Mix. The thermal cycling conditions for all PCRs were: 1 cycle of 95°C for 2 min (polymerase activation); 40 cycles at 95°C for 5 s (melting), followed by 60°C for 20 s (annealing and signal acquisition). For melting curve analysis, the default setting was used: rise 1°C each step from 72 to 95°C, wait for 90 s on the first step of the pre-melting condition and then wait for 5 s for each step afterward. When PCR cycle threshold (Ct) value is less than 30 at melting temperature of the amplification products *T*_*m*_ = 84.06 ± 0.21°C, the sample was considered as positive.

**TABLE 1:** Primers used in this study.

**Primer name**	**Sequence**	**Product size (bp)**
MCR-1/2/6-F	GTCGTCGGTGAGACGGC	198
MCR-1/2/6-R	GTATTTGGCGGTATCGACATCA	
MCR-3/7-F	AACACATGCTATGACGAGGTTGT	228
MCR-3/7-R	GGTGTAGCGGATGGTGTTGTC	
MCR-4-F	TGCGAAGAATGCCAGTCGTA	169
MCR-4-R	GCCGCATGAGCTAGTATCGT	
MCR-5-F	TGCGCAACTACGGGGTTTAT	328
MCR-5-R	CGAATGCCCGAGATGACGTA	
MCR-8-F	CCTGCATGTTCTCGCGAATG	486
MCR-8-R	GCATCCCGGAATAACGTTGC	

PCR-positive cultures were plated on TSA plates containing 2 μg/mL of colistin. Candidate colistin-resistant colonies (5–10) were picked from plates with an appropriate serial dilution (∼ 50 colonies/plate) and dipped into a microcentrifuge tube with 50 μL of water. After boiling for 10 min, 2 μL of the lysate was further analyzed by PCR to confirm the presence of the *mcr* gene. *mcr*-positive strains were preserved in TSB containing 30% glycerol and stored at −80°C.

### Whole Genome Sequencing

Genomic DNA from *mcr*-positive strains were extracted for single-molecule real-time (SMRT) sequencing using the Blood and Cell Culture DNA Midi kit (Qiagen cat. no. 13343, CA) and Genomic DNA Buffer Set (Qiagen cat. no. 19060, CA), according to manufacturer’s protocols for preparation of Gram-negative bacteria sample and isolation of genomic DNA from bacteria. The 20-kb DNA libraries were prepared using the BluePippin size selection system following manufacturer’s instructions. High-throughput sequencing was performed on a PacBio RSII platform using the 360-min data collection protocol. The PacBio reads were assembled using the Hierarchical Genome Assembly Process 3 (HGAP3, SMRT Analysis v2.3.0). The completed genome sequences were submitted to NCBI Prokaryotic Genome Annotation Pipeline (PGAP) for annotation.

### Species Identification and Genetic Analysis of *mcr*-positive Strains

The multilocus phylogenetic analysis (MLPA) was used to identify the species of each *Aeromonas* strain based on seven housekeeping genes, including *gyrB, rpoD, gyrA, recA, dnaJ, dnaX*, and *atpD* (Martinez-Murcia et al., 2011). Strains isolated in this study, along with 25 other *Aeromonas* strains were analyzed by MLPA. Phylogenetic tree was constructed based on the concatenated sequences in the same order using MEGA 5.0 and maximum likelihood method with 1000 bootstrap replicates. The phylogenetic trees of *mcr-3* variants, *mcr-3-*like genes, *mcr-3*-related phosphoethanolamine-lipid A transferase coding genes and *mcr-7*-related phosphoethanolamine-lipid A transferase coding genes were also constructed using MEGA 5.0 and maximum likelihood method with 1000 bootstrap replicates. Alignment of gene clusters was performed using Mauve (version snapshot_2015-02-25) and Easyfig (version 2.2.2) (Darling et al., 2004; Sullivan et al., 2011). Searching of antimicrobial resistance genes in the whole genome sequence of each strain was performed by ABRicate^1^ against the comprehensive antibiotic resistance database (CARD).

### Antimicrobial Susceptibility Testing

The minimum inhibitory concentration (MIC) of colistin for each strain was determined using the broth microdilution (BMD) method recommended by the Clinical and Laboratory Standards Institute (CLSI). Briefly, BMD is performed using cation-adjusted Mueller-Hinton broth, a range of 2-fold dilutions of colistin (ranging from 0.25 to 128 mg/L), and a bacterial inoculum density of 500,000 cfu/mL per well. *E. coli* strains, AR-Bank #0346 (MIC of colistin = 4 mg/L) and AR-Bank #0349 (MIC of colistin = 2–4 mg/L), were used as positive controls, and *E. coli* reference strain, ATCC25922, was used as a negative control. Resistant breakpoint for colistin was adopted from CLSI with MIC ≤ 2 mg/L as the susceptibility breakpoint and MIC > 2 mg/L as the resistance breakpoint.

### Nucleotide Sequence Accession Number

The whole-genome nucleotide sequences of 14 *Aeromonas* strains have been submitted to DDBJ/EMBL/GenBank. The accession numbers were listed in Table 2.

**TABLE 2:** Some characteristics of *mcr-3/7*-positive *Aeromonas* strains isolated in this study.

**Isolate**	**Isolate source**	**Species**	***mcr* variant and *mcr-3*-like gene**	**Colistin MIC (mg/L)**	**Accession No.**
1805	Catfish	*A. hydrophila*	*mcr-3-related*	128	CP038515
2359	Chicken rinse	*A. hydrophila*	*mcr-3-related*	128	CP043324
2692	Chicken rinse	*A. hydrophila*	*mcr-3.27, mcr-3-like1, mcr-3-related*	128	CP038513-CP038514
2961	Chicken rinse	*A. hydrophila*	*mcr-3-related*	128	VHIX00000000
3019	Catfish	*A. hydrophila*	*mcr-3-related*	128	CP053885
3036	Chicken rinse	*A. jandaei*	*mcr-3-related, mcr-7-related*	2	CP053882
3206	Chicken rinse	*A. hydrophila*	*mcr-3-related*	128	CP043323
3299	Chicken rinse	*A. jandaei*	*mcr-3-related, mcr-7-related*	128	CP043322
3384	Chicken rinse	*A. jandaei*	*mcr-3-related, mcr-7-related*	128	CP043321
3924	Chicken rinse	*A. hydrophila*	*mcr-3-related*	128	CP053884
4484	Chicken rinse	*A. hydrophila*	*mcr-3.27, mcr-3-like2, mcr-3-related*	128	VHIW00000000
4608	Poultry	*A. jandaei*	*mcr-3, mcr-3-like3, mcr-3-related, mcr-7-related*	128	CP053881
4956	Chicken rinse	*A. jandaei*	*mcr-3-related, mcr-7-related*	128	CP053879-CP053880
4960	Chicken rinse	*A. hydrophila*	*mcr-3-related*	128	CP053883

## Results

### Prevalence of *mcr*-positive Bacteria in U.S. Beef, Pork, Poultry, and Catfish

The prevalence of *mcr* in U.S. food-producing animal samples was investigated by enrichment culture and real-time PCR using a total of 5,169 samples, including 1,787 chicken rinse, 1,369 ground beef, 1,057 beef trim, 416 pork, 363 poultry, and 177 catfish. One pork sample was identified to be PCR-positive for *mcr-1/2/6*, and 14 samples were found to be PCR-positive for *mcr*-3/7. A *mcr-1*-positive *Escherichia coli* strain, EC2492, was isolated from the pork sample that was PCR-positive for *mcr-1/2/6* and characterized phenotypically and genomically in details (Wang Y. et al., 2020). Fourteen *mcr-3/7*-positive isolates were identified from the corresponding samples that were positive by PCR. Table 2 shows some basic information about these isolates. The prevalence of *mcr-3/7* were 0.28% (1/363) in poultry, 1.13% (2/177) in catfish, and 0.62% (11/1787) in chicken rinse, respectively. The overall prevalence of *mcr-3/7* was 0.27% in all meat samples tested. No other *mcr* genes, including *mcr-2*, *mcr-4*, *mcr-5*, *mcr-6*, and *mcr-8* were found in this survey.

### Identification and Genomic Characterization of *mcr*-3/7-positive Isolates

**FIGURE 1:**
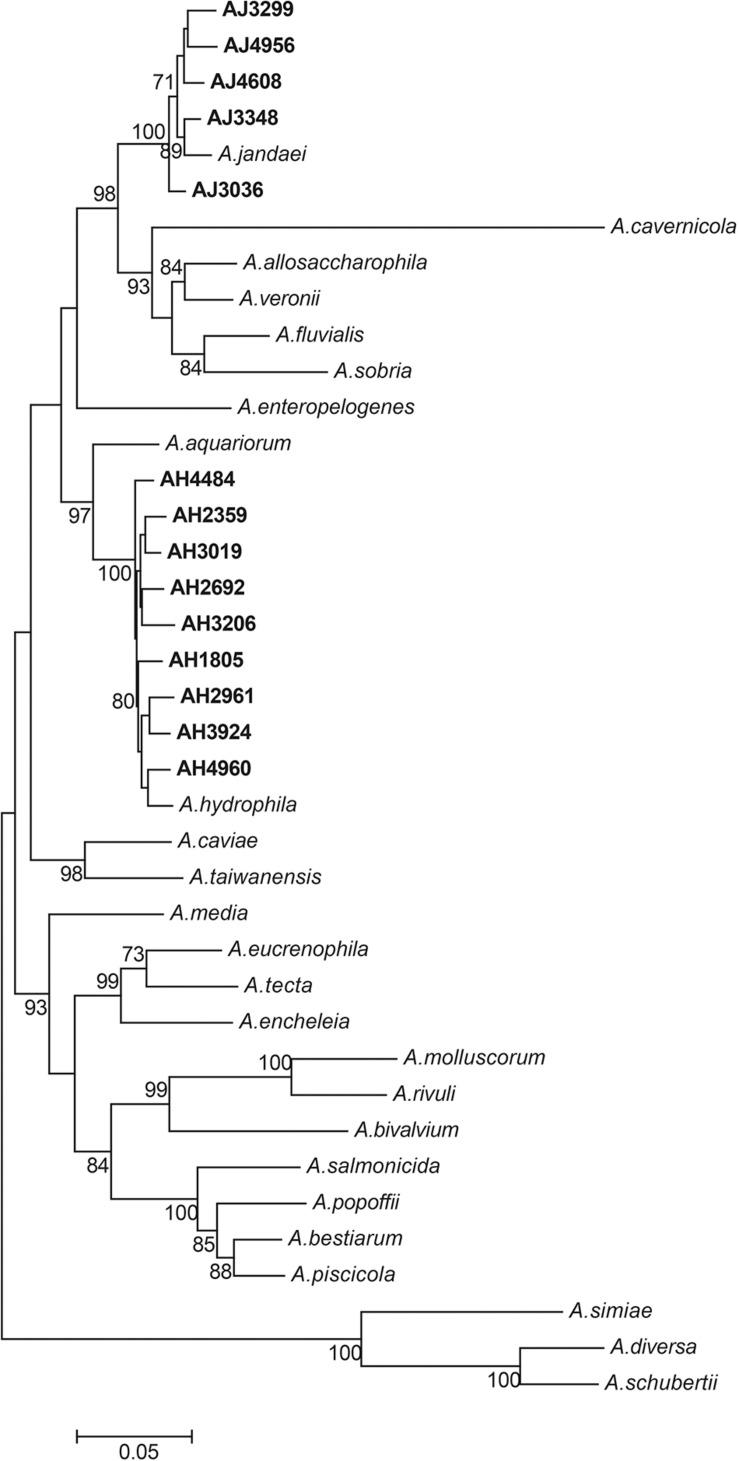
Phylogenetic tree constructed using maximum likelihood method based on the concatenate sequences of seven housekeeping genes from 14 *Aeromonas* strains isolated in this study and 25 publicly available *Aeromonas* strains. The strains isolated in this study were written in bold font.

All 14 *mcr-3/7*-positive isolates were identified as *Aeromonas* species based on results from BLAST searching the database of 16S ribosomal RNA sequences. Nine strains, including AH1805, AH2359, AH2692, AH2961, AH3019, AH3206, AH3924, AH4484, and AH4960, were identified as *Aeromonas hydrophila*, and five strains, including AJ3036, AJ3299, AJ3384, AJ4608, and AJ4956, were identified as *Aeromonas jandaei* based on the MLPA (Figure 1). The genomes of six *A. hydrophila* strains were assembled into one scaffold with sizes varied from 4.8 Mb to 5.2 Mb and the GC contents varied from 60.96 to 61.58%. The genome of strain AH2692 was assembled into two scaffolds, including one 5.0 Mb chromosome and one 10 kb plasmid. The genome of strain AH2961 was assembled into three scaffolds, including one 4.8 Mb-chromosome and two plasmids with sizes of 35 and 12 kb in length, respectively. The genome of strain AH4484 was assembled into 12 scaffolds with a total length of 4.8 Mb and average GC content of 61.63%. The GC contents of the five *A. jandaei* strains varied from 58.87 to 59.01% and the genomes of four were assembled into one scaffold with sizes varied from 4.5 to 4.6 Mb, and one (AJ4956) was assembled into two scaffolds, including a 4.5 Mb-chromosome and an 147 kb-plasmid with a GC content of 55.4%.

### *mcr*-3-*mcr*-3-like Segment in *Aeromonas* Strains

**FIGURE 2:**
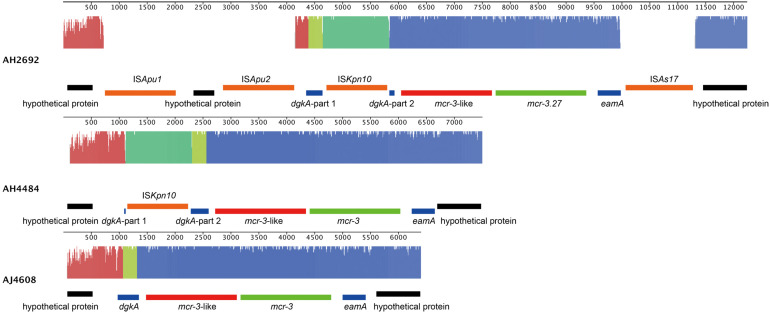
Alignment and genomic organization of *mcr-3-mcr-3-*like clusters in *A. hydrophila* strains, AH2692, AH4484, and *A. jandaei* strain AJ4608. The annotation information is shown above or under each gene block.

**FIGURE 3:**
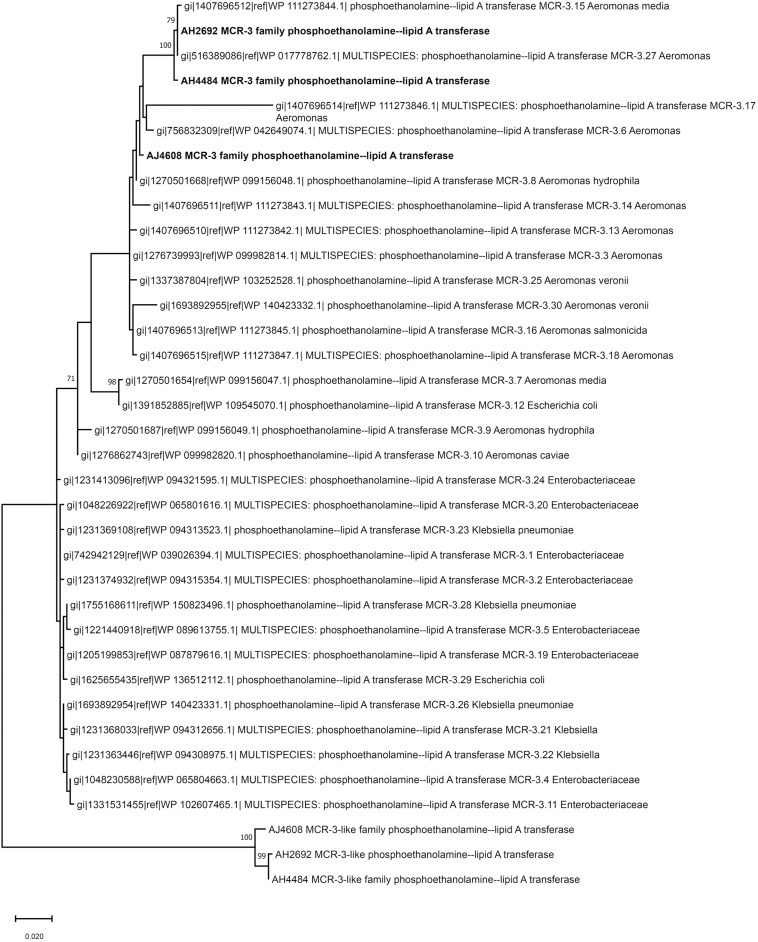
Phylogenetic tree constructed based on amino acid sequences of *mcr-3* variants, *mcr-3-*like genes identified in this study and *mcr-3* variants available in public database.

Two *A. hydrophila* strains, AH2692 and AH4484, and one *A. jandaei* strain, AJ4608, harbored a *mcr-3-mcr-3-*like segment, which was flanked by an *eamA* gene (EamA family transporter) and a *dgkA* gene (diacylglycerol kinase). The *dgkA* genes in strains AH2692 and AH4484 were interrupted by the IS*Kpn10* family transposase genes at different positions. An IS element, IS*As17*, was found upstream of the *eamA* gene in strain AH2692, just like what was found in an *A. dhakensis* strain isolated from human peritoneal fluid (accession no. AOBN01000008.1). In addition, two IS4 family transposase genes, IS*Apu1* and IS*Apu2* were found downstream of the *mcr-3-mcr-3-*like segment (Figure 2). Unlike in *A. hydrophilla* strains, no IS elements were present adjacent to the *mcr-3-mcr-3-*like segment in strain AJ4608. The *mcr*-3 variant gene in strain AH2692 was 1623 bp long and coded for a protein of 540 amino acids that was identical to the MCR-3.27 (WP_017778762.1). The *mcr-3* variant gene in strain AH4484 was 1557 bp long and coded for a protein of 518 amino acids that was almost identical to the MCR-3.27 except for missing the first 22 amino acids and having 2 amino acid substitutions. The *mcr-3* gene in strain AJ4608 was 1623 bp long and coded for a protein of 540 amino acids that was 100% identical to MCR-3, a phosphoethanolamine-lipid A transferase (WP_118854326.1) from an *Aeromonas veronnni* strain and highly similar to MCR-3.8 (WP_099156048.1) with 2 amino acid substitutions. The three *mcr-3*-like genes adjacent to the *mcr-3* genes were more divergent to all reported *mcr-3* gene variants (Figure 3). The insertion regions harboring the *mcr-3-mcr-3-*like cluster, were located between genes *rimO* (ribosomal protein S12 methylthiotransferase) and HD domain-containing protein coding gene with sizes of 52 and 30 kb in strain AH2692 and AH4484, respectively. Additionally, the insertion region of strain AH2692 harbored a Tn3 family transposon associated with mercuric resistance. In strain AJ4608, the insertion region harboring the *mcr-3-mcr-3-like* cluster was 36 kb long and inserted in a *ligA* (DNA ligase) gene.

### *mcr*-3-related Genes in *Aeromonas* Strains

**FIGURE 4:**
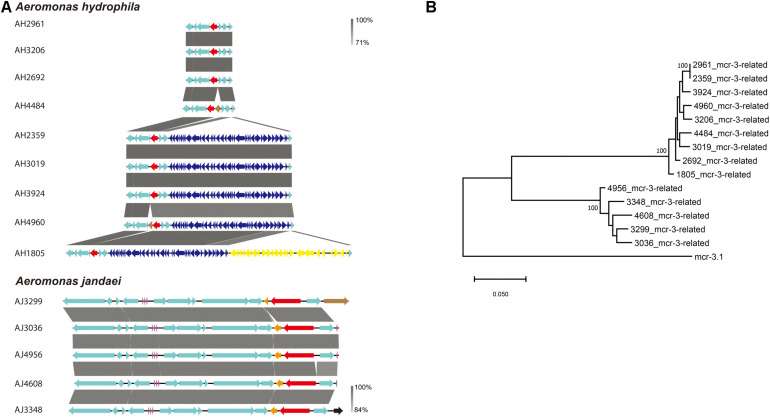
**(A)**. Alignment of *mcr-3*-related phosphoethanolamine-lipid A transferase coding gene in *A. hydrophila* and *A. jandaei* strains isolated in this study. The levels of nucleotide identity were shown in gray shaded regions by color intensity. The *mcr-3*-related genes were marked in red. The transposase genes were marked in brown. tRNAs were marked in purple. The type III secretion system were showed in dark blue and the gene cluster associated with tellurium resistance were showed in yellow. **(B)**. Phylogenetic tree of *mcr-3*-related gene, along with the reference sequence of *mcr-3.1*.

A *mcr-3*-related gene encoding for an MCR-3-related phosphoethanolamine-lipid A transferase was identified in all *Aeromonas* strains isolated in this study. The genetic environment of *mcr-3*-related genes in *A. hydrophila* strains consisted of upstream genes for a MFS (Major Facilitator Superfamily) transporter and a hypothetical protein and downstream genes for another hypothetical protein, a MprF (Multiple Peptide Resistance Factor) and a virulence factor (Figure 4A). Additionally, there was a sequence coding for a type III secretion system inserted between a tRNA and a MFS transporter gene in strains AH1805, AH2359, AH3019, AH3924, and AH4960. In strain AH1805, there was another gene cluster associated with tellurium resistance next to the sequence for a type III secretion system (Figure 4A). The genetic environment of *mcr-3*-related genes in *A. jandaei* strains consisted of an upstream gene for a hypothetical protein, and downstream genes for a DGKA, a virulence factor and a MprF protein (Figure 4A). Notably, all the *mcr-3* related genes present in *A. hydrophila* clustered into one genetic group, while all the *mcr-3* related genes present in *A. jandaei* clustered into another distinct group. Compared with the deduced amino acid sequence of *mcr-3.1*, the *mcr-3*-related genes shared 70.65–70.96% identity in *A. hydrophila*, and 72.74–73.42% identity in *A. jandaei* (Figure 4B).

### *mcr*-7-related Genes in *A. jandaei* Strains

**FIGURE 5:**
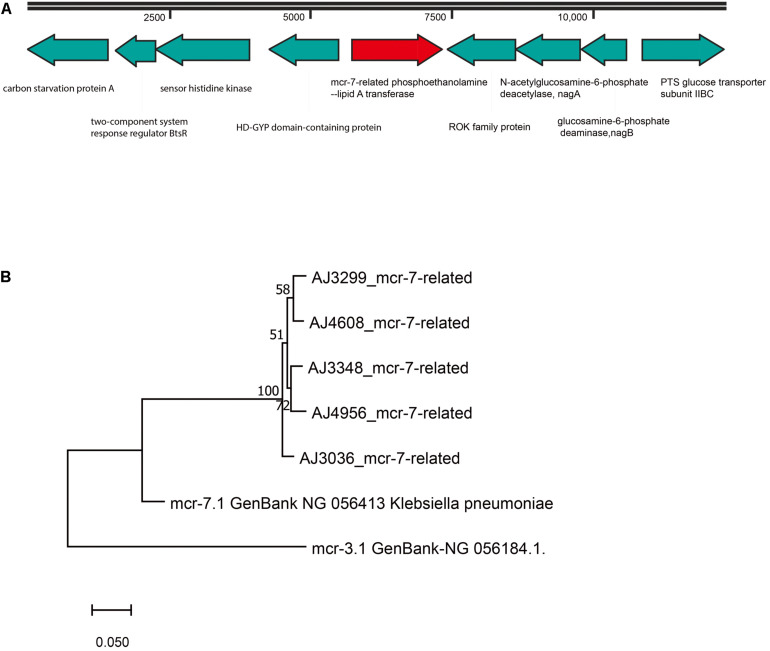
**(A)**. Genetic environment of *mcr-7*-related phosphoethanolamine-lipid A transferase coding gene in *A. jandaei* strains. **(B)**. Phylogenetic tree of *mcr-7*-related gene, along with the reference sequences of *mcr-7.1* and *mcr-3.1*.

A *mcr-7-*related gene encoding for a MCR-7-related phosphoethanolamine-lipid A transferase was found in all *A. jandaei* strains. The *mcr-7*-related genes in *A. jandaei* shared 81.79–82.34% identity to *mcr-7.1* at nucleic acid level. Figure 5A shows genes in flanking regions of the *mcr-7*-related gene, which were conserved in all *A. jandaei* strains. Figure 5B shows the phylogenetic tree of *mcr-7-*related genes in *A. jandaei* strains and the *mcr-7.1* gene, with the *mcr-3.1* gene as an outgroup (Figure 5B).

### Colistin Susceptibility Test

Based on results from BMD tests, all 14 *mcr-3/7*-positive *Aeromonas* strains were resistant to colistin. Except for strain AJ3036 that showed a borderline resistance to colistin (MIC = 2 mg/L), all other 13 strains exhibited very high resistance to colistin with MIC ≥ 128 mg/L.

### Other Antimicrobial Resistance Genes

In addition to colistin resistance genes, a variety of other antimicrobial resistance genes (ARGs) involved in resistance to cephalosporin, carbapenem, aminoglycoside, and sulfonamide were identified in 14 colistin-resistant *Aeromonas* isolates (Table 3). The *bla*_OXA–12_ and *bla*_OXA–724_ genes, encoding Oxacillin-hydrolyzing (OXA)-type β-lactamases for resistance to cephalosporin, were observed in all *A. jandaei* and *A. hydrophila* strains. Another three ARGs, *tru-1, cepS*, and *aqu-2* responsible for resistance to cephalosporin were found in one, five, and four *Aeromonas* strains, respectively. Four *cphA* variant genes, including *cphA2*, *cphA4*, *cphA7*, and *cphA8*, were identified in 5 *A. hydrophila* and 3 *A. jandaei* strains. These *cphA* genes code for carbapenem-hydrolyzing metallo-β-lactamases responsible for resistance to carbapenem. The *imiH* and *ceph-A3* genes accounting for resistance to carbapenem were found in three *A. hydrophila* and two *A. jandaei* strains, respectively. A strain (AH2359) harboring six ARGs responsible for resistance to cephalosporins, carbapenems, aminoglycosides, and sulfonamides was identified. All ARGs mentioned above were located on the chromosome.

**TABLE 3:** Other antimicrobial resistance genes identified using the comprehensive antibiotic resistance database (CARD).

**ARG**	**Resistance**	**AH1805**	**AH2359**	**AH2692**	**AH2961**	**AH3019**	**AJ3036**	**AH3206**	**AJ3299**	**AJ3384**	**AH3924**	**AH4484**	**AJ4608**	**AJ4956**	**AH4960**
*bla_OXA–12_/ bla_OXA–724_*	cephalosporin	+	+	+	+	+	+	+	+	+	+	+	+	+	+
*tru-1*	cephalosporin	-	-	-	-	-	-	-	-	-	-	-	+	-	-
*cepS*	cephalosporin	+	+	+	-	-	-	+	-	-	+	-	-	-	-
*aqu-2*	cephalosporin	-	-	-	+	-	-	-	-	+	-	+	-	-	+
*imiH*	carbapenem	-	+	-	+	-	-	-	-	-	-	+	-	-	-
*cphA*	carbapenem	-	-	+	-	+	+	+	+	+	+	-	-	-	+
*ceph-A3*	carbapenem	-	-	-	-	-	-	-	-	-	-	-	+	+	-
*ant(3″)-IIa*	aminoglycoside	-	+	-	-	-	-	-	-	-	-	-	-	-	-
*aac(3)-VIa*	aminoglycoside	-	+	-	-	-	-	-	-	-	-	-	-	-	-
*sul1*	sulfonamide	-	+	-	-	-	-	-	-	-	-	-	-	-	-

## Discussion

This is the second report on the prevalence of *mcr* genes in U.S. domestic animal-origin samples following our first report (Wang Y. et al., 2020). Among 5,169 samples, one *E. coli* strain carrying a plasmid-borne *mcr-1* gene was isolated from a raw pork sample. The characteristics of the strain was described, previously (Wang Y. et al., 2020). Here, we report 14 chromosome-borne *mcr*-positive strains isolated from 1 poultry, 2 catfish and 11 chicken rinse samples. No *mcr-*positive samples were found in ground beef or beef trim samples. Based on data obtained from this survey, the prevalence of chromosome-borne *mcr* (0.27%) was much higher than that of plasmid-borne *mcr* (0.02%) in the U.S. animal-derived samples.

It was found that all 14 *mcr*-positive strains belonged to *Aeromonas.* Among them, nine *A. hydrophila* strains harbored a *mcr-3*-related gene, while five *A. jandaei* strains harbored a *mcr-3*-related gene plus a *mcr-7*-related gene. In addition, a *mcr-3-mcr-3-*like segment was present in three *Aeromonas strains*, including AH2692, AH4484, and AJ4608. This *mcr-3-mcr-3-*like segment was originally reported in *A. veronni* 172 isolated from chicken meat (accession no. KY924928.1) (Ling et al., 2017). Subsequently, the *mcr-3.6-mcr-3-*like, *mcr-3.8-mcr-3-*like, and *mcr-3.9-mcr-3-*like segments were reported in *Aeromonas allosaccharophila*, *A. jandaei*, and *A. hydrophila*, respectively (Eichhorn et al., 2018). The widespread of *mcr-3* genes in *Aeromonas* species suggests that *Aeromonas* may be the origin of *mcr-3* genes (Shen et al., 2018). In this study, a variety of complete or truncated IS elements, including IS*AS17*, IS*Kpn10*, IS*Apu1*, and IS*Apu2* were identified in proximity to the *mcr-3.27-mcr-3*-like segments in strains AH2692 and AH4484, suggesting these IS elements may play a role in the mobility of these colistin resistance genes. However, no IS elements, were identified near the *mcr-3-mcr-3-*like segment in strain AJ4608. It has been reported that a *dgkA* gene, coding for a diacylglycerol kinase involving in the phosphatidic acid pathway, was frequently present immediately downstream of *mcr* genes in both chromosome and plasmid (Ling et al., 2017; Yin et al., 2017). In-depth analysis of the flanking regions, a *dgkA* gene was found downstream of the *mcr-3-mcr-3-*like segments in AH2692, AH4484, and AJ4608 strains and downstream of the *mcr-3* related genes in 5 *A. jandaei* strains. In addition, an IS element with 91% nucleotide identity to IS*Aeme13* (IS4 family) was located next to a hypothetical protein immediately upstream of the *mcr-3*-related gene in *A. jandaei* strain AJ3299. It is possible that this IS element involves in transferring *mcr-3* genes between chromosome and plasmid. As *Aeromonas* species are routine microflora in poultry and fish, the presence of potentially transferrable *mcr* genes in *Aeromonas* could facilitate the spread of these genes to other bacteria species living in the same habitat.

It was reported that *A. hydrophila* had low level of resistance to colistin but showed increased MIC values following preculture with low dose of colistin, while *A. jandaei* exhibited intrinsic resistance to colistin (Fosse et al., 2003). A research from 479 unrelated *Aeromonas* isolates showed that only 0.84% of *Aeromonas* strains carried a *mcr* gene, suggesting that *mcr* is not the intrinsic genes in *Aeromonas* (Eichhorn et al., 2018). In this study, almost all *Aeromonas* strains (13 out of 14) isolated showed much higher resistance to colistin (MICs ≥ 128 mg/L) than the *Escherichia coli* strain WJ1 (MIC = 8 mg/L) and *Klebsiella pneumoniae* strain SC20141012 (MIC = 4 mg/L), the original strains reported to harbor the plasmid-borne *mcr*-3.1 and *mcr*-7.1, respectively (Yin et al., 2017; Yang et al., 2018). Whether the high level of colistin resistance is due to a synergistic effect from *mcr* and other ARG genes remains unknown. Surprisingly, strain AJ3036, an *Aeromonas* species with innate resistance to colistin showed borderline resistance to colistin, although it carried genes responsible for colistin-resistance, including *mcr-3*- and *mcr-7*-related genes. The correlation between *mcr-3-/mcr-7*-related genes and colistin resistance, or the machinery on neutralizing the resistance to colistin in this strain is awaiting to be elucidated.

Based on the whole genome sequences, ten ARGs involving resistance to four kinds of antimicrobials were identified in 14 *Aeromonas* strains. The OXA-type β-lactamases that belong to class D β-lactams and are responsible for much of the β-lactams like penicillin and cephalosporins resistance are widely identified in *Aeromonas* species. Consistently, the *bla*_OXA–12_ and *bla*_OXA–724_ (also named *ampH*) genes, originated from *A. jandaie* AER 14 and *A. hydrophila* T429125 strains, respectively, were found in all 14 *Aeromonas* isolates (Rasmussen et al., 1994; Avison et al., 2000). Carbapenems are usually regarded as the most effective antibiotics for serious infections, however, their usage was compromised by the appearance of carbapenemases.

In conclusion, a total of 14 *mcr-3/7*-positive *Aeromonas* strains with high level of colistin resistance were isolated in a study of 5,169 food-producing animal samples collected by FSIS. Results based on whole genome sequences of these isolates indicated that *A. hydrophila* strains only carried *mcr-3* genes on their chromosome, while *A. jandaie* strains carried both chromosome-borne *mcr-3* and *mcr-7* genes. A set of *mcr-3-mcr-3-*like segment was identified in two *A. hydrophila* and one *A. jandaie* isolates. A variety of IS elements were observed in proximity to some *mcr-3/7* genes. In accordance with other studies (Ling et al., 2017; Shen et al., 2018), our findings further confirmed that *mcr-3/7* are more common in *Aeromonas* than in other bacterial species although they are not intrinsic genes in *Aeromonas*. In addition to *mcr* genes, 10 other ARGs involving in resistance to cephalosporin, carbapenem, aminoglycoside and sulfonamide were found in these strains. Of note, one *A. hydrophila* strain harbored six ARGs responsible for four types of antimicrobial resistance, simultaneously. Food contamination with colistin-resistance genes that have the potential to transfer among different bacterial species both vertically and horizontally could pose huge risk to animal and human health, global surveillance and collaborations are needed to combat or prevent such threat.

## Data Availability Statement

The datasets presented in this study can be found in online repositories. The names of the repository/repositories and accession number(s) can be found in the article/Supplementary Material.

## Author Contributions

XH and YW contributed to the conception and design of the work, ensuring any part of the work are appropriately investigated and resolved, critically revised the final version to be published, and were responsible for the integrity of the work. NH, YG, YW, and RR contributed by performing sequencing, analyzing the data, and revising the manuscript. All authors contributed to the article and approved the submitted version.

## Conflict of Interest

The authors declare that the research was conducted in the absence of any commercial or financial relationships that could be construed as a potential conflict of interest.

## Footnotes

1 https://github.com/tseemann/abricate

## References

[B1] AbuOun M. Stubberfield E. J. Duggett N. A. Kirchner M. Dormer L. Nunez-Garcia J. (2018). mcr-1 and mcr-2 (mcr-6.1) variant genes identified in *Moraxella* species isolated from pigs in Great Britain from 2014 to 2015. *J. Antimicrob. Chemother.* 73:2904. 10.1093/jac/dky272 30053008 PMC6148207

[B2] Avison M. B. Niumsup P. Walsh T. R. Bennett P. M. (2000). Aeromonas hydrophila AmpH and CepH beta-lactamases: derepressed expression in mutants of *Escherichia coli* lacking creB. *J. Antimicrob. Chemother.* 46 695–702. 10.1093/jac/46.5.695 11062187

[B3] Borowiak M. Fischer J. Hammerl J. A. Hendriksen R. S. Szabo I. Malorny B. (2017). Identification of a novel transposon-associated phosphoethanolamine transferase gene, mcr-5, conferring colistin resistance in d-tartrate fermenting *Salmonella enterica* subsp. enterica serovar Paratyphi B. *J. Antimicrob. Chemother.* 72 3317–3324. 10.1093/jac/dkx327 28962028

[B4] Carattoli A. Villa L. Feudi C. Curcio L. Orsini S. Luppi A. (2017). Novel plasmid-mediated colistin resistance mcr-4 gene in *Salmonella* and *Escherichia coli*, Italy 2013, Spain and Belgium, 2015 to 2016. *Euro Surveill.* 22:30589. 10.2807/1560-7917.ES.2017.22.31.30589 28797329 PMC5553062

[B5] Carroll L. M. Gaballa A. Guldimann C. Sullivan G. Henderson L. O. Wiedmann M. (2019). Identification of novel mobilized colistin resistance gene mcr-9 in a multidrug-resistant, colistin-susceptible *Salmonella enterica* serotype typhimurium isolate. *mBio* 10:e00853-19. 10.1128/mBio.00853-19 31064835 PMC6509194

[B6] Darling A. C. Mau B. Blattner F. R. Perna N. T. (2004). Mauve: multiple alignment of conserved genomic sequence with rearrangements. *Genome Res.* 14 1394–1403. 10.1101/gr.2289704 15231754 PMC442156

[B7] Eichhorn I. Feudi C. Wang Y. Kaspar H. Fessler A. T. Lubke-Becker A. (2018). Identification of novel variants of the colistin resistance gene mcr-3 in *Aeromonas* spp. from the national resistance monitoring programme GERM-Vet and from diagnostic submissions. *J. Antimicrob. Chemother.* 73 1217–1221. 10.1093/jac/dkx538 29394397

[B8] Falagas M. E. Rafailidis P. I. (2008). Re-emergence of colistin in today’s world of multidrug-resistant organisms: personal perspectives. *Expert Opin. Investig. Drugs* 17 973–981. 10.1517/13543784.17.7.973 18549335

[B9] Fosse T. Giraud-Morin C. Madinier I. (2003). Induced colistin resistance as an identifying marker for *Aeromonas* phenospecies groups. *Lett. Appl. Microbiol.* 36 25–29. 10.1046/j.1472-765x.2003.01257.x 12485337

[B10] Ling Z. Yin W. Li H. Zhang Q. Wang X. Wang Z. (2017). Chromosome-Mediated mcr-3 variants in *Aeromonas veronii* from Chicken Meat. *Antimicrob. Agents Chemother.* 61:e01272-17. 10.1128/AAC.01272-17 28848017 PMC5655048

[B11] Liu Y.-Y. Wang Y. Walsh T. R. Yi L.-X. Zhang R. Spencer J. (2016). Emergence of plasmid-mediated colistin resistance mechanism MCR-1 in animals and human beings in China: a microbiological and molecular biological study. *Lancet Infect. Dis.* 16 161–168. 10.1016/s1473-3099(15)00424-7 26603172

[B12] Luo Q. Wang Y. Xiao Y. (2020). Prevalence and transmission of mobilized colistin resistance (mcr) gene in bacteria common to animals and humans. *Biosaf. Health* 2 71–78. 10.1016/j.bsheal.2020.05.001

[B13] Martinez-Murcia A. J. Monera A. Saavedra M. J. Oncina R. Lopez-Alvarez M. Lara E. (2011). Multilocus phylogenetic analysis of the genus *Aeromonas*. *Syst. Appl. Microbiol.* 34 189–199. 10.1016/j.syapm.2010.11.014 21353754

[B14] Rasmussen B. A. Keeney D. Yang Y. Bush K. (1994). Cloning and expression of a cloxacillin-hydrolyzing enzyme and a cephalosporinase from Aeromonas sobria AER 14M in *Escherichia coli*: requirement for an *E. coli* chromosomal mutation for efficient expression of the class D enzyme. *Antimicrob. Agents Chemother.* 38 2078–2085. 10.1128/aac.38.9.2078 7811022 PMC284687

[B15] Shen Y. Xu C. Sun Q. Schwarz S. Ou Y. Yang L. (2018). Prevalence and genetic analysis of mcr-3-positive *Aeromonas* species from humans, retail meat, and environmental water samples. *Antimicrob. Agents Chemother.* 62 e404–e418. 10.1128/AAC.00404-18 29967026 PMC6125509

[B16] Sullivan M. J. Petty N. K. Beatson S. A. (2011). Easyfig: a genome comparison visualizer. *Bioinformatics* 27 1009–1010. 10.1093/bioinformatics/btr039 21278367 PMC3065679

[B17] Wang C. Feng Y. Liu L. Wei L. Kang M. Zong Z. (2020). Identification of novel mobile colistin resistance gene mcr-10. *Emerg. Microbes Infect.* 9 508–516. 10.1080/22221751.2020.1732231 32116151 PMC7067168

[B18] Wang X. Wang Y. Zhou Y. Li J. Yin W. Wang S. (2018). Emergence of a novel mobile colistin resistance gene, mcr-8, in NDM-producing *Klebsiella pneumoniae*. *Emerg. Microbes Infect.* 7:122. 10.1038/s41426-018-0124-z 29970891 PMC6030107

[B19] Wang Y. Hou N. Johnston J. Sarreal C. Jarosh J. Hughes A. C. (2020). Low prevalence of mobile colistin-resistance in U.S. meat, catfish, poultry and genomic characterization of a mcr-1 positive *Escherichia coli* strain. *Food Control* 118:107434. 10.1016/j.foodcont.2020.107434

[B20] Xavier B. B. Lammens C. Ruhal R. Kumar-Singh S. Butaye P. Goossens H. (2016). Identification of a novel plasmid-mediated colistin-resistance gene, mcr-2, in *Escherichia coli*, Belgium, June 2016. *Euro Surveill.* 21 10.2807/1560-7917.ES.2016.21.27.30280 27416987

[B21] Yang Y. Q. Li Y. X. Lei C. W. Zhang A. Y. Wang H. N. (2018). Novel plasmid-mediated colistin resistance gene mcr-7.1 in *Klebsiella pneumoniae*. *J. Antimicrob. Chemother.* 73 1791–1795. 10.1093/jac/dky111 29912417

[B22] Yin W. Li H. Shen Y. Liu Z. Wang S. Shen Z. (2017). Novel Plasmid-mediated colistin resistance gene mcr-3 in *Escherichia coli*. *mBio* 8:e00543-17. 28655818 10.1128/mBio.00543-17 PMC5487729

