# Supplementary material for: Prevalence and Genetic Analysis of Chromosomal mcr-3/7 in Aeromonas From U.S. Animal-Derived Samples
Source: Front Microbiol. 2021 Apr 30;12:667406. doi: 10.3389/fmicb.2021.667406 (PMC8120114; doi:10.3389/fmicb.2021.667406)
Supplement: Supplementary file 1 [file Table_1.docx]

Table S1 Seven housekeeping genes of 27 *Aeromonas* strains used in Multi-locus Phylogenetic Analysis

| **Strain** | **Species name** | **GenBank Locus** | | | | | | |
| --- | --- | --- | --- | --- | --- | --- | --- | --- |
|  |  | ***atpD*** | ***dnaJ*** | ***dnaX*** | ***gyrA*** | ***gyrB*** | ***recA*** | ***rpoD*** |
| MDC 12 | *A. allosaccharophila* | HQ442563 | HQ443062 | HQ442458 | HQ443157 | HQ442732 | HQ442964 | HQ442836 |
| MDC 317 | *A. aquariorum* | HQ442588 | HQ443056 | HQ442484 | HQ443171 | HQ442717 | HQ442909 | HQ442802 |
| MDC 34 | *A. bestiarum* | HQ442544 | HQ442987 | HQ442431 | HQ443099 | HQ442684 | HQ442973 | HQ442857 |
| CECT 7113 | *A. bivalvium* | HQ442557 | HQ443036 | HQ442527 | HQ443141 | HQ442703 | HQ442882 | HQ442817 |
| MDC 2508 | *A. cavernicola* | HQ442573 | HQ443080 | HQ442426 | HQ443164 | HQ442702 | HQ442978 | HQ442864 |
| CECT 838 | *A. caviae* | HQ442602 | HQ443008 | HQ442422 | HQ443146 | HQ442748 | HQ442921 | HQ442790 |
| MDC 2583 | *A. diversa* | HQ442647 | HQ443085 | HQ442535 | HQ443195 | HQ442757 | HQ442873 | HQ442806 |
| MDC 64 | *A. encheleia* | HQ442630 | HQ443030 | HQ442493 | HQ443137 | HQ442654 | HQ442886 | HQ442780 |
| MDC 89 | *A. enteropelogenes* | HQ442599 | HQ443043 | HQ442487 | HQ443189 | HQ442720 | HQ442931 | HQ442820 |
| MDC 256 | *A. eucrenophila* | HQ442617 | HQ443018 | HQ442512 | HQ443119 | HQ442661 | HQ442894 | HQ442773 |
| LMG 24681 | *A. fluvialis* | HQ442605 | FJ603454 | HQ442464 | FJ603456 | FJ603455 | FJ603457 | FJ603453 |
| MDC 48 | *A. hydrophila* | HQ442562 | HQ443045 | HQ442474 | HQ443177 | HQ442742 | HQ442928 | HQ442795 |
| MDC 581 | *A. jandaei* | HQ442575 | HQ443073 | HQ442454 | HQ443181 | HQ442739 | HQ442914 | HQ442838 |
| MDC 219 | *A. media* | HQ442612 | HQ443014 | HQ442503 | HQ443133 | HQ442705 | HQ442976 | HQ442784 |
| MDC 72 | *A. molluscorum* | HQ442638 | HQ443004 | HQ442520 | HQ443114 | HQ442669 | HQ442879 | HQ442815 |
| MDC 2516 | *A. piscicola* | HQ442547 | HQ442991 | HQ442433 | HQ443101 | HQ442692 | HQ442952 | HQ442860 |
| MDC 30 | *A. popoffii* | HQ442553 | HQ442997 | HQ442440 | HQ443106 | HQ442695 | HQ442945 | HQ442849 |
| MDC 2512 | *A. rivuli* | HQ442642 | FJ969441 | HQ442525 | FJ969438 | FJ969439 | FJ969440 | FJ969437 |
| MDC 25 | *A. salmonicida* | HQ442538 | HQ442980 | HQ442443 | HQ443091 | HQ442679 | HQ442959 | HQ442846 |
| MDC 574 | *A. schubertii* | HQ442650 | HQ443086 | HQ442531 | HQ443196 | HQ442753 | HQ442874 | HQ442807 |
| MDC 2374 | *A. simiae* | HQ442645 | HQ443083 | HQ442530 | HQ443193 | GQ860942 | HQ442871 | GQ860943 |
| MDC 103 | *A. sobria* | HQ442570 | HQ443078 | HQ442449 | HQ443149 | HQ442700 | HQ442939 | HQ442868 |
| LMG 24683T | *A. taiwanensis* | HQ442603 | FJ807270 | HQ442491 | FJ807274 | FJ807272 | FJ807273 | FJ472928 |
| MDC 93 | *A. tecta* | HQ442624 | HQ443021 | HQ442499 | HQ443124 | HQ442664 | HQ442896 | HQ442764 |
| MDC 57 | *A. veronii* | HQ442584 | HQ443067 | HQ442465 | HQ443162 | HQ442727 | HQ442968 | HQ442835 |
